# Research on Mechanism of Fatigue Life Enhancement in Ball Bearings by Residual Compressive Stress

**DOI:** 10.3390/ma19153321

**Published:** 2026-08-05

**Authors:** Ruijie Xie, Wenhu Zhang, Jun Xu, Jianbo Xu, Yiping Xu

**Affiliations:** 1 School of Mechanical and Electrical Engineering, Henan University of Science and Technology, Luoyang 471000, China; 9906112@haust.edu.cn; 2 Zhongzhe High-Speed Railway Bearing Co., Ltd., Quzhou 324000, China; xujun773939187@163.com; 3 Hangzhou Dishichuan Intelligent Equipment Co., Ltd., Hangzhou 310000, China; 18668188008@163.com; 4 Aviation Industry Changhe Aircraft Industry (Group) Co., Ltd., Jingdezhen 333000, China; 13979882086@163.com

**Keywords:** fatigue life, residual compressive stress, finite element analysis, improvement mechanism, ball bearing

## Abstract

**Highlights:**

- The first FE model with depth-gradient residual stress reveals subsurface fatigue mechanism in bearings.
- RCS shifts shear stress depth and orientation, impeding crack initiation and propagation.
- The critical RCS depth for optimal performance is identified, even under high friction.

**Abstract:**

While the beneficial effect of residual compressive stress (RCS) on rolling bearing fatigue life is empirically well-established, the underlying mechanisms, particularly its influence on the subsurface stress field responsible for fatigue initiation, remain inadequately explored. This study employs a sophisticated finite element (FE) model of a ball–raceway contact, which incorporates a depth-dependent gradient of RCS, to elucidate the underlying mechanisms. The results demonstrate that RCS not only reduces the contact stress at the interface but also fundamentally alters the subsurface stress field by shifting the location of the maximum shear stress to a greater depth and reducing its inclination angle. These changes collectively delay crack initiation and propagation, explaining the observed enhancement in fatigue life. Furthermore, the study demonstrates that the beneficial effect of RCS is depth-dependent and persists significantly even under high-friction conditions.

## 1. Introduction

The residual stress state within the bearing surface layer significantly influences rolling contact fatigue life. As stress cycles accumulate during operation, the residual stress evolves, leading to complex and non-uniform fatigue phenomena [1]. In recent years, operating conditions of rolling bearings have increasingly exceeded conventional design limits, imposing stricter requirements on service life. Multiple factors affect bearing fatigue life, including material properties, clearance, surface roughness, lubrication, and surface treatments [2,3,4]. In this paper, by adjusting the contact parameters, the contact analysis with clearance becomes a highly nonlinear problem. The solver needs continuous iterative calculation to determine when the parts contact and the extent of the contact force. This process is costly can easily lead to solution failure. When the initial state is set as contact (zero clearance), the solver establishes the contact relationship from the beginning, which greatly reduces the degree of nonlinearity and makes the calculation more stable and efficient.

Extensive research has been conducted on bearing fatigue life. Huang Jian [5] discussed the effect of material composition, showing that trace element addition can significantly improve fatigue performance. Littman [6,7,8] addressed the role of working clearance and its selection based on actual conditions to extend service life. Abdullah [9,10] investigated the influence of surface roughness, revealing that increased roughness heightens raceway stress and can lead to premature fatigue. Kumagai [11] proposed carbonitriding as an effective strengthening technique to extend bearing life. Furthermore, studies [12] indicate that residual stress present after heat treatment affects dynamic performance. This residual stress, arising from inhomogeneous plastic deformation during manufacturing [13,14], exists in equilibrium without external loads.

The authors of one study [15] employed the finite element method to analyze the formation of residual stress in bearing rings. Measurement techniques are generally categorized as destructive or non-destructive, with the blind-hole and X-ray diffraction methods being widely used [16]. Various studies have used X-ray analysis to measure the depth-wise distribution of residual stress in raceway subsurfaces, showing average compressive stresses of between 266 and 438 MPa [17,18,19]. Liu Xiao [20] established an FE model showing that RCS enhances contact stiffness. Andrić [21,22,23] found that tensile residual stress increases contact stress and promotes surface damage, while Junshuai [24,25,26] concluded that residual stress influences subsurface crack initiation speed and direction. Numerous experiments confirm that spalling often results from shear-driven crack propagation from the subsurface to the surface [27,28,29].

Additionally, residual tensile stress can mitigate stress concentration at notches, thereby delaying crack growth and influencing the applied stress amplitude [29,30,31,32]. While prior studies have confirmed that residual stress distribution affects rolling contact behavior, the mechanism by which RCS improves fatigue life remains unclear. This paper investigates the effect of RCS on bearing contact behavior, focusing on the depth and orientation of the maximum shear stress, and elucidates the mechanism of fatigue life enhancement. Compared with experimental approaches, numerical simulation efficiently addresses engineering challenges while providing reliable data for subsequent experimental validation [33]. Therefore, a robust simulation model is indispensable for a comprehensive analysis of the effect of RCS on overall bearing life.

Compared with the traditional research mentioned above, this article will elaborate on the specific mechanism by which RCS improves the fatigue life of bearings and explore the influence of RCS on bearing contact behavior, which has important engineering significance. The paper is structured as follows: First, a finite element model incorporating near-surface residual stress is established. Second, the influence of RCS on contact performance and crack propagation is analyzed. Third, a specific ball bearing model is used to simulate residual stress distribution, comparing fatigue life with and without residual stress. Finally, the mechanism by which RCS affects bearing fatigue life is discussed.

## 2. Model and Validation

### 2.1. Method

**Figure 1:**
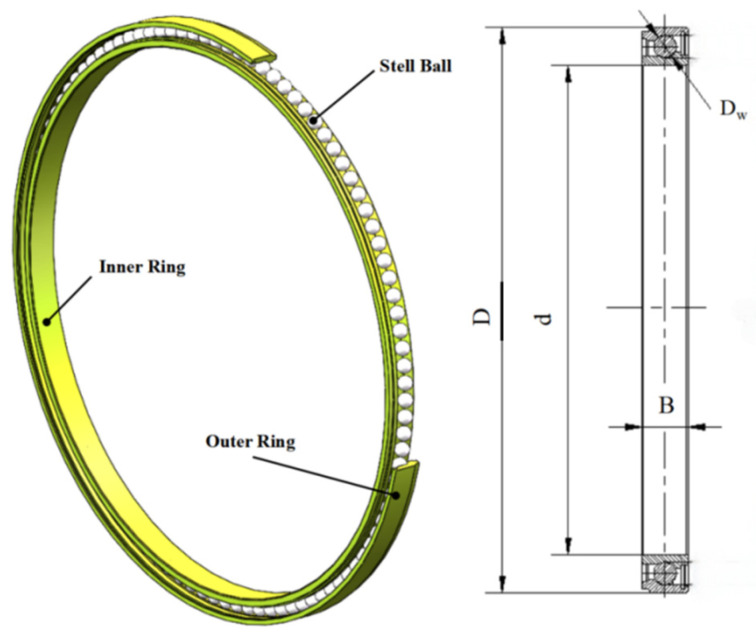
3D model of the bearing.

This study investigates an angular contact ball bearing. Material parameters of bearing components are shown in Table 1. The three-dimensional (3D) model of the bearing is illustrated in Figure 1 and its key geometric parameters are listed in Table 2. The rollers, inner, and outer rings are made of GCr15 bearing steel, which was modeled as a linearly elastic and isotropic material. The bearing examined in the present work is a key structural element within the helicopter propeller assembly. The bearing examined in the present work is a key structural element within the helicopter propeller assembly. By investigating the influence of residual stress on bearing fatigue life from a microscopic perspective, this study aims to establish a quantitative link between micro-structural features and macroscopic durability. Extending the fatigue life of such a critical component is of significant importance for enhancing the overall performance and reliability of the helicopter.

**Figure 2:**
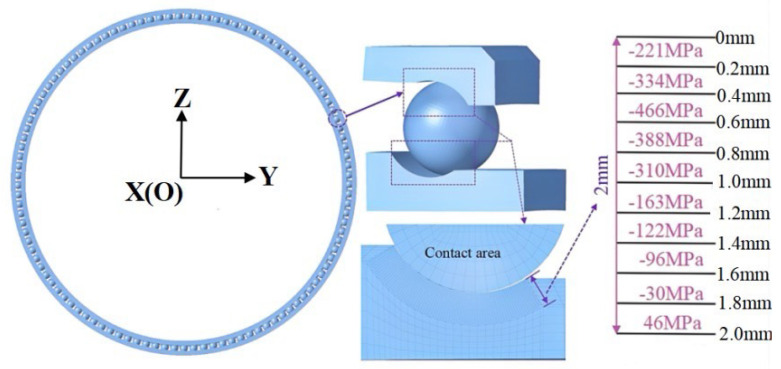
Finite element model with residual stress distribution.

The finite element contact model is illustrated in Figure 2. The mesh was generated using HyperWorks (2022), and the simulations were performed in PERMAS (V21). The model comprises 4646942 elements, with a refined size of 0.2 mm in the contact regions and a coarser size of 1.5 mm elsewhere. SOLID186 elements were used for the bearing components, and CONTA174 elements defined the contact pairs between the ball and the raceways. A dedicated subsurface zone (2 mm deep, divided into ten 0.2 mm thick layers) was created to implement the residual stress gradient.

The boundary conditions were applied using rigid coupling points. All nodes on the outer ring’s outside surface were coupled to Point 1, which was fully constrained. All nodes on the inner ring’s bore and side faces were coupled to Point 2, which was constrained radially but subjected to a prescribed axial displacement along the X-axis (bearing axis).

As shown in Figure 2, the Z-axis corresponds to the radial direction. Unlike traditional uniform assumptions, a multi-layer gradient approach was employed to enhance the physical fidelity of residual stress modeling [26]. A RCS profile, representative of surface enhancement treatments like shot peening [23], was applied to the subsurface layers. The specific stress values, which decay approximately linearly with depth, are provided in Figure 2. This 3D gradient model enables a more accurate analysis of the influence mechanism compared to conventional studies [25].

The finite element analysis process of this paper is as follows: the finite element analysis model is established through SOILDWORKS (2022), the preprocessing mesh of the model is divided through HyperMesh, and the model is analyzed and solved by using the nonlinear contact solution software PERMAS (V21).

### 2.2. Model Validation

The operational conditions used for model validation are listed in Table 3.

In Condition 1, in the range of common bearing steel (elastic modulus fluctuation range ≤ 5%), the influence weight of material mechanical property parameters on the peak contact stress is about 3~5%. Compared with the load fluctuation (influence weight of about 60~70%) and the friction coefficient (influence weight of about 20%), the influence of the change in material properties on the final fatigue life prediction results is in a secondary position.

The finite element analysis (FEA) clarifies the role of subsurface RCS in contact performance. To validate the model, the contact pressures from FEA were compared against theoretical analytical (TA) solutions based on Hertzian contact theory [34,35]. As shown in Table 4, the maximum error between FEA and TA results is 2.5%, which confirms the accuracy of the finite element model.

A mesh sensitivity study was conducted to ensure the results were independent of the discretization. Contact stresses were computed using mesh sizes of 0.05, 0.1, 0.2, 0.4, and 0.6 mm in the critical contact zone. The results are summarized in Table 5. The solution becomes unstable when the mesh size is coarsened beyond 0.2 mm, as evidenced by the significant increase in calculated contact pressure. This indicates that a mesh size of 0.2 mm or finer is required for accurate results.

The Grid Convergence Index (GCI) [36] was calculated for the finer meshes to quantitatively assess discretization error. The GCI is computed as

(1) $$\mathrm{GCI}=F_{s}\frac{\left|\epsilon \right|}{r^{p}-1}$$

where F_s_ is a safety factor, r is the grid refinement ratio, p is the order of convergence, and ϵ is the relative error. For example, the GCI between the 0.1 mm and 0.2 mm meshes was below 5%, a threshold indicating acceptable mesh independence [37]. Considering both computational efficiency and result accuracy, a mesh size of 0.2mm was selected for all subsequent simulations.

## 3. Results and Discussions

The axial displacement of the inner ring under extreme working conditions in Table 3 is 1 mm. To investigate the effect of RCS on fatigue life, the bearing was subjected to axial displacements ranging from 0.2 mm to 1.0 mm, as listed in Table 6. This loading method was employed because it ensures a uniform load distribution among all rolling elements. Since each ball carries an identical load under axial displacement, the contact behavior of a single ball–inner raceway pair is analyzed and discussed below to elucidate the fundamental mechanism of residual stress.

### 3.1. Effects of RCS on Contact Stress

**Figure 3:**
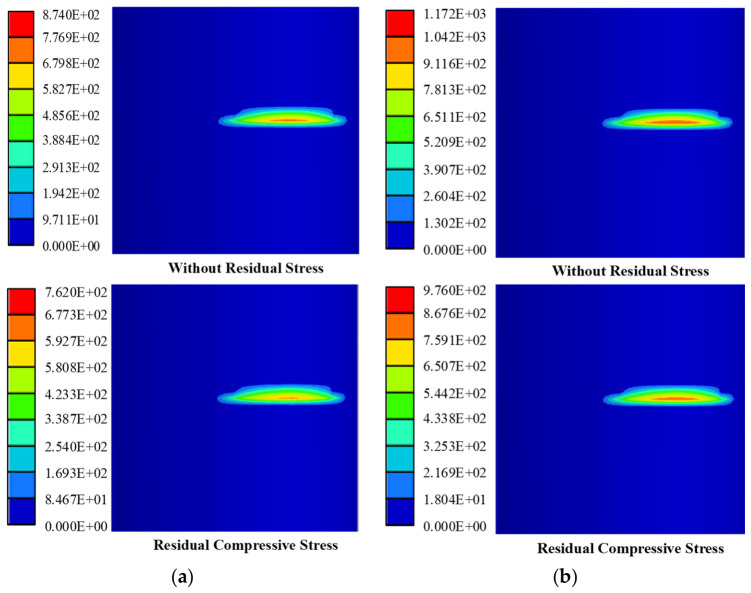

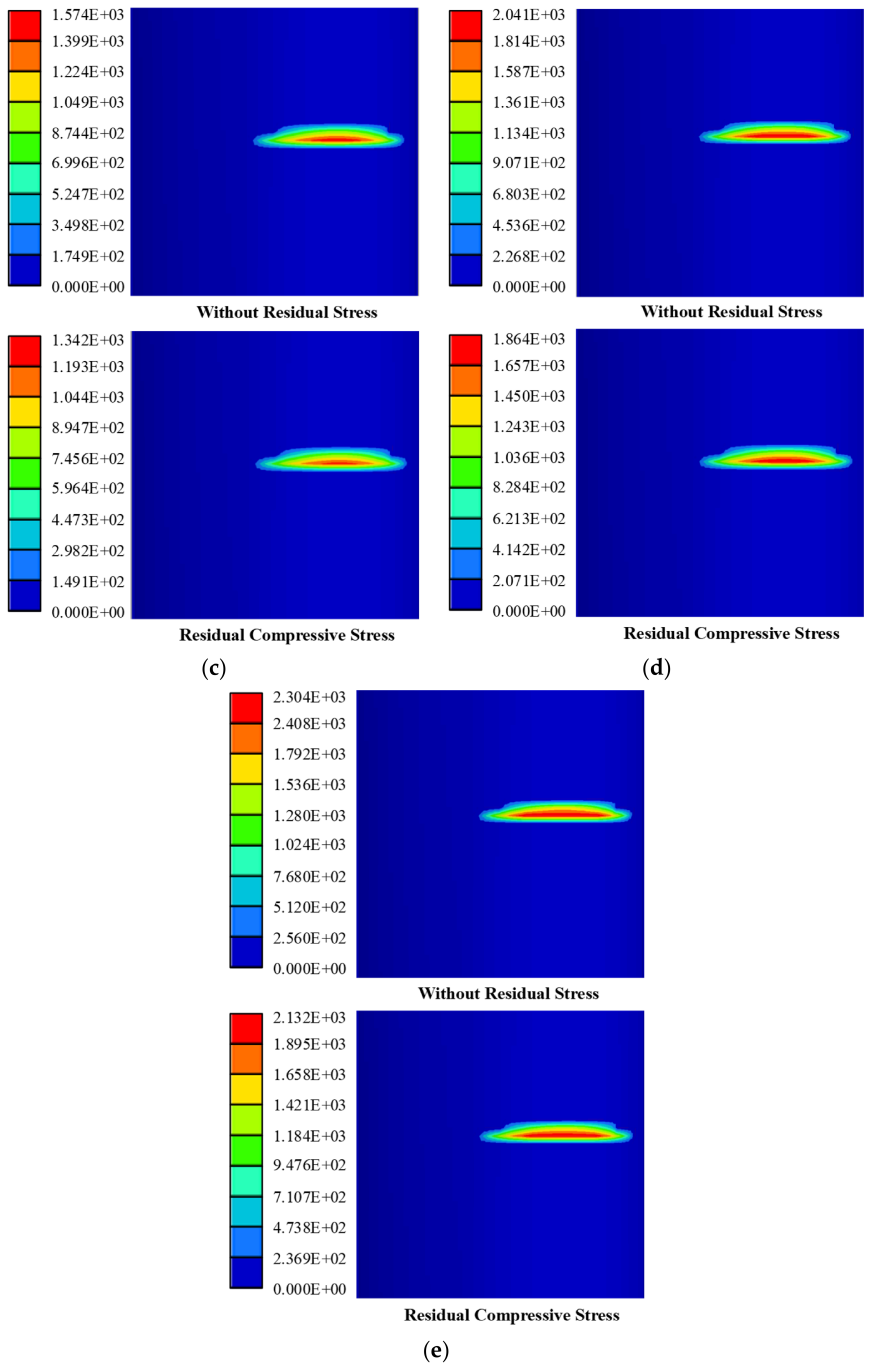
Contact pressure distribution at the ball–inner raceway interface (MPa). (**a**) 0.2 mm; (**b**) 0.4 mm; (**c**) 0.6 mm; (**d**) 0.8 mm; (**e**) 1.0 mm.

**Figure 4:**
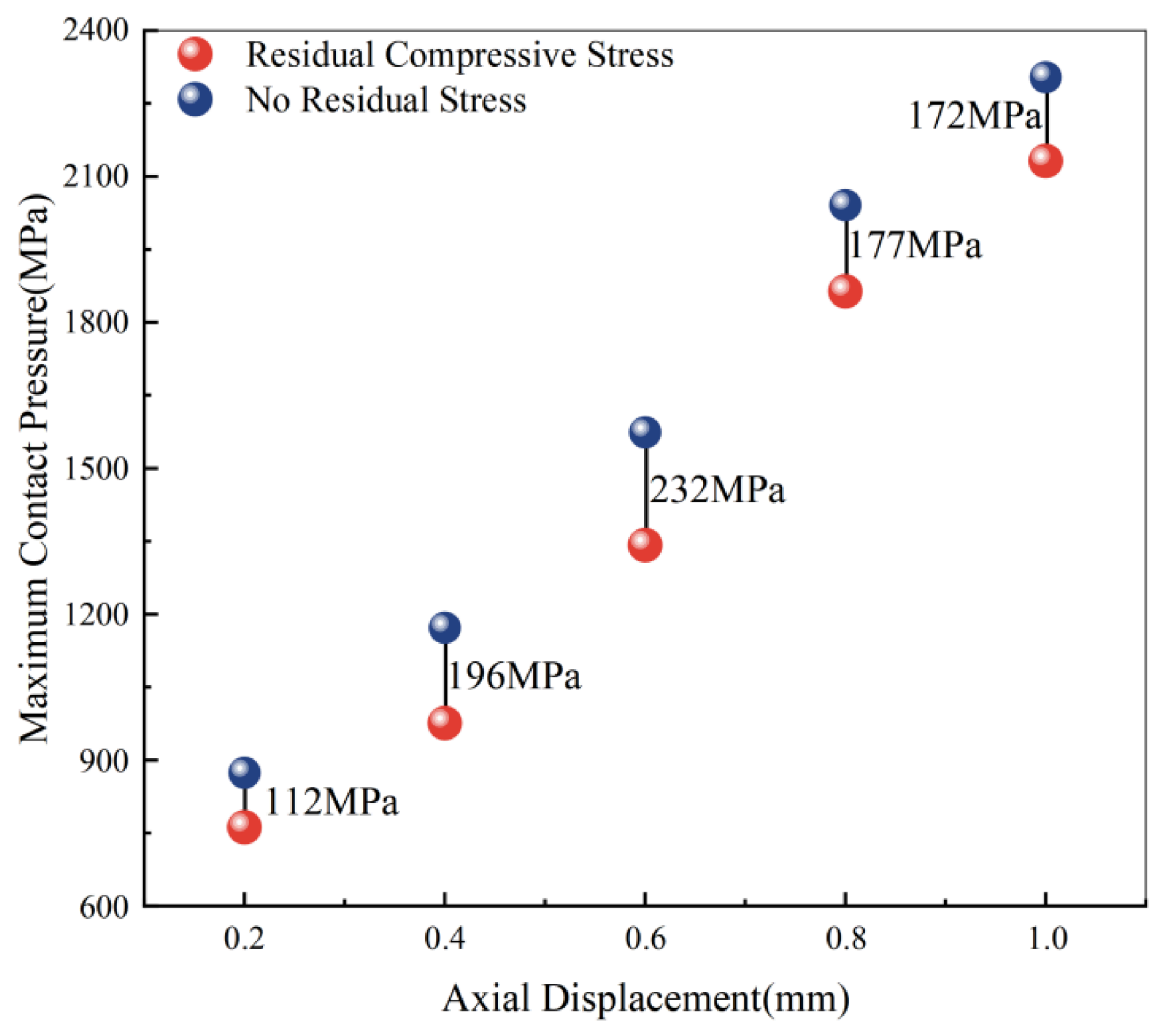
Maximum contact pressure versus axial displacement.

**Figure 5:**
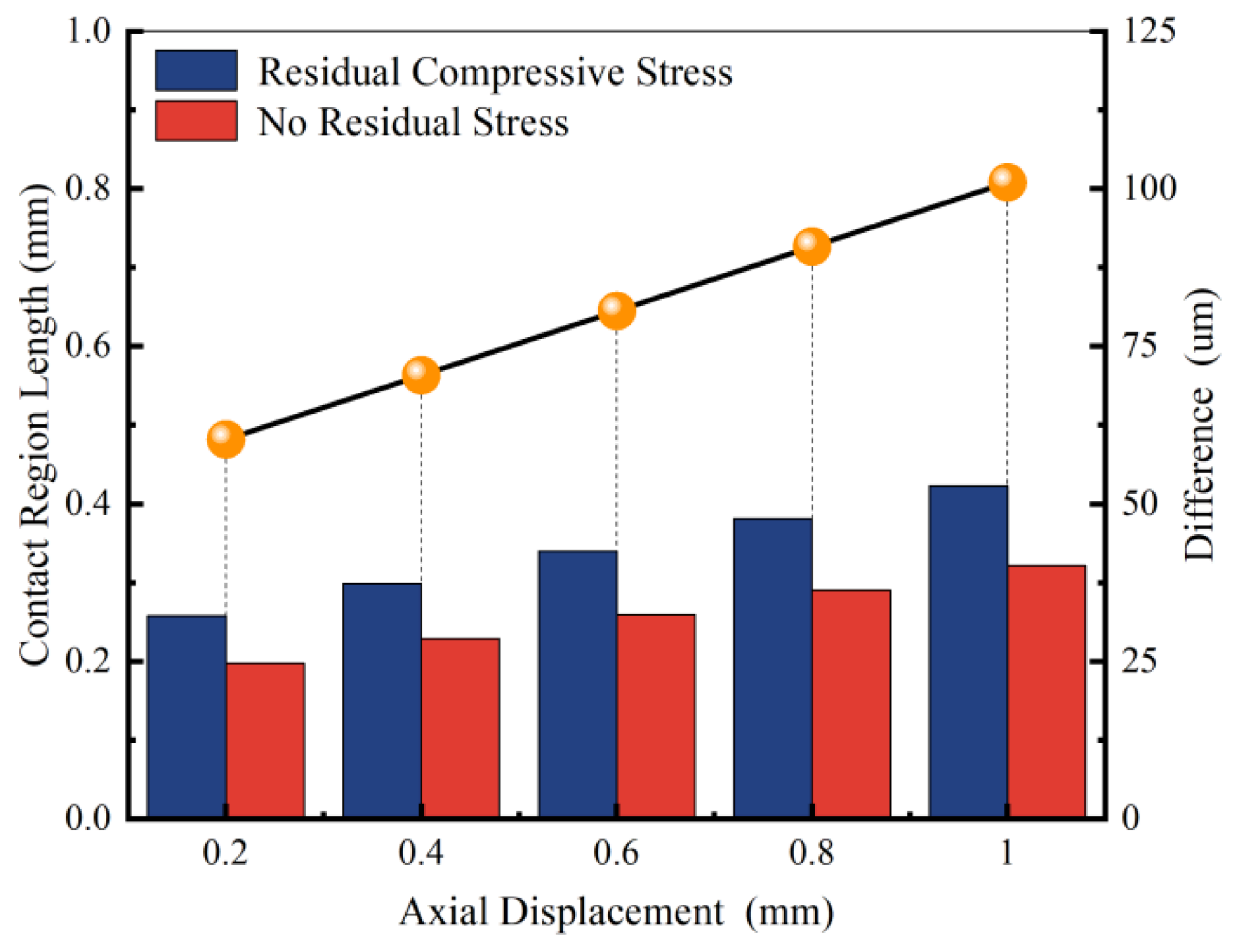
Length of the major axis of the contact ellipse versus axial displacement.

The contact pressure distribution between the ball and inner raceway, with and without RCS, is compared in Figure 3. The corresponding maximum contact pressure and the length of the contact ellipse’s major axis across different axial displacements are quantified in Figure 4 and Figure 5, respectively.

The results demonstrate that the presence of RCS consistently reduces both the maximum contact pressure and the dimensions of the contact ellipse across all investigated loading conditions. For instance, at an axial displacement of 0.6 mm (Case 3), the presence of RCS reduces the peak contact pressure by approximately 232 MPa and shortens the major axis by 0.12 mm.

**Figure 6:**
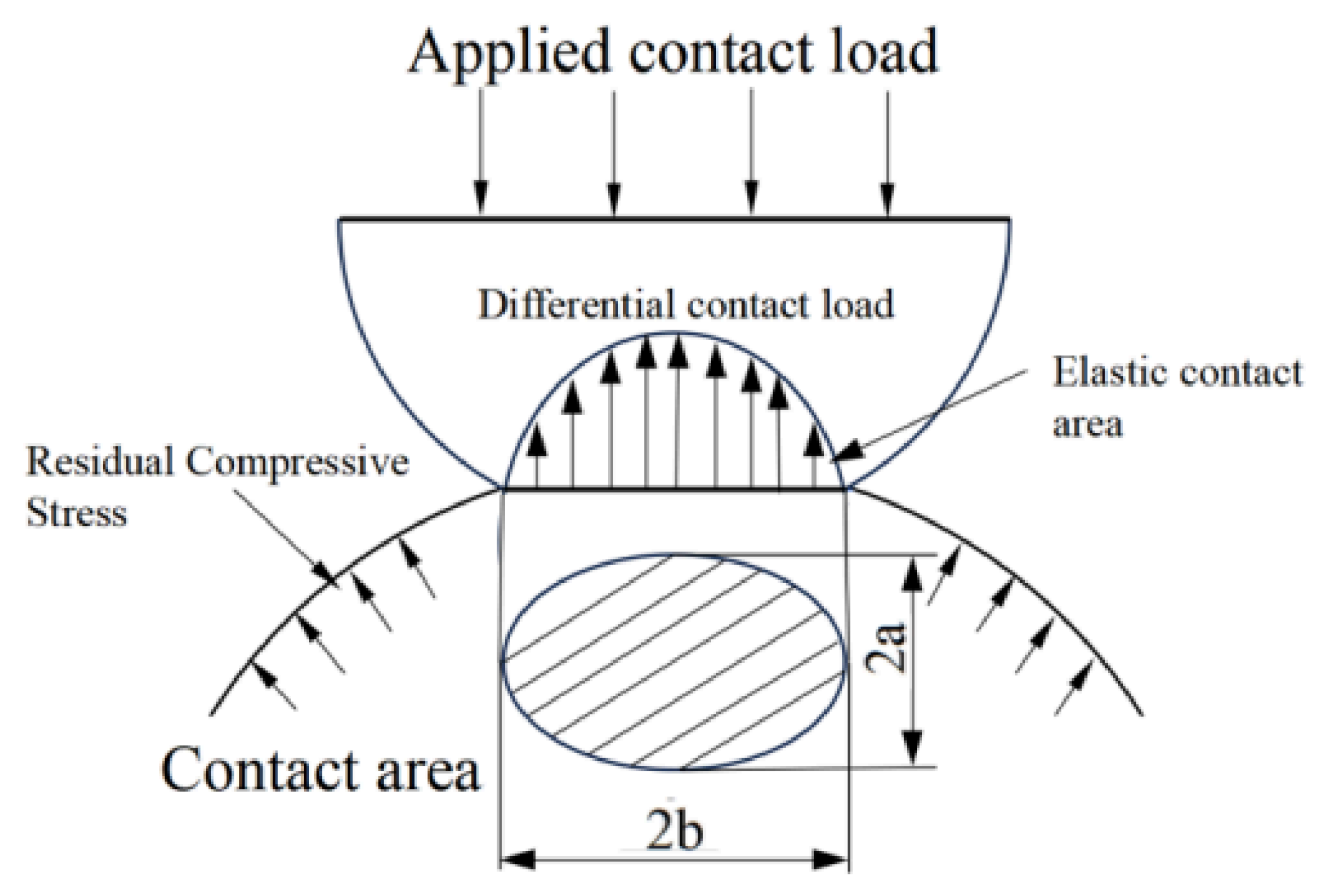
RCS mechanism.

The underlying mechanism for this reduction is illustrated in Figure 6. The pre-existing RCS in the raceway subsurface superimposes a normal force that opposes the applied contact load. This superposition effectively offsets a portion of the externally applied load, leading to a lower resultant contact stress and a smaller contact area.

### 3.2. Influence of RCS on Maximum Shear Stress

#### 3.2.1. Depth of Maximum Shear Stress

**Figure 7:**
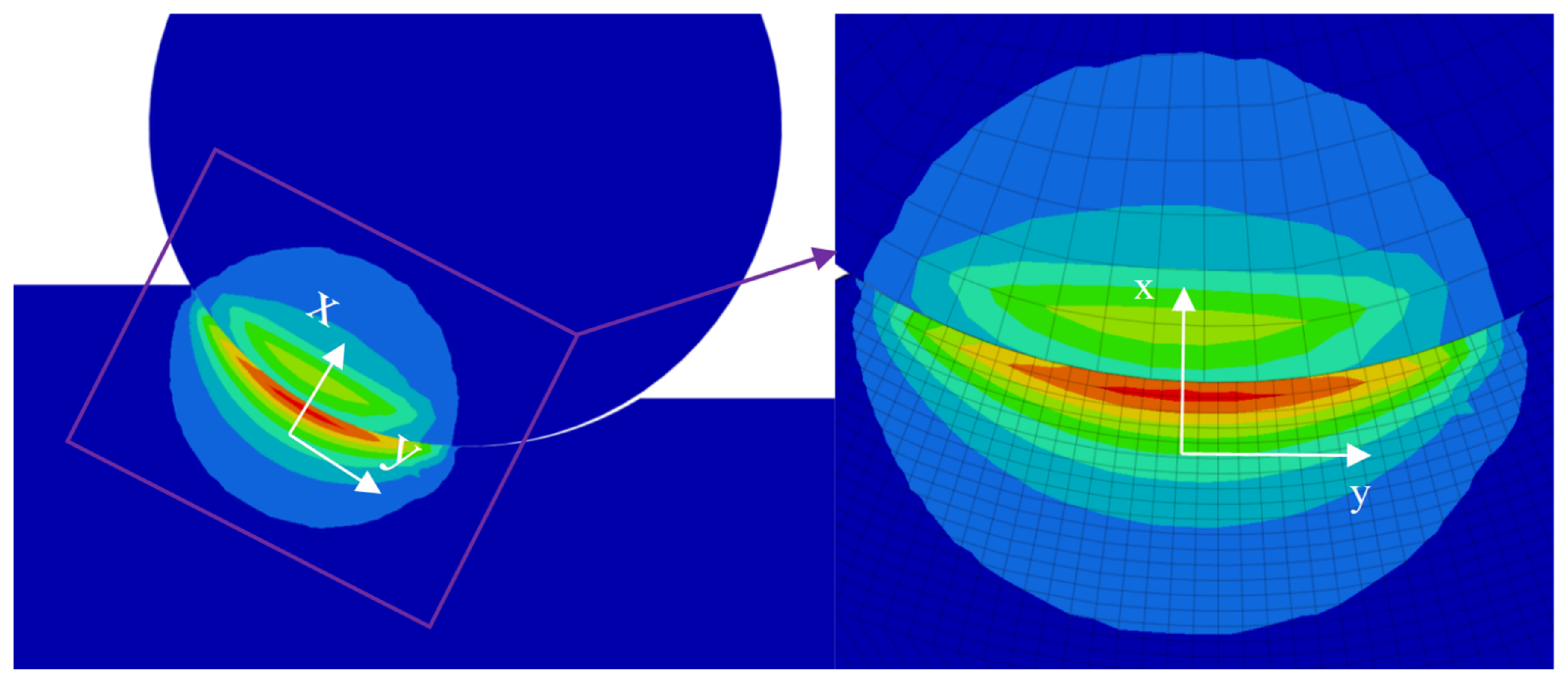
Shear stress distribution in the raceway subsurface (qualitative contour).

**Figure 8:**
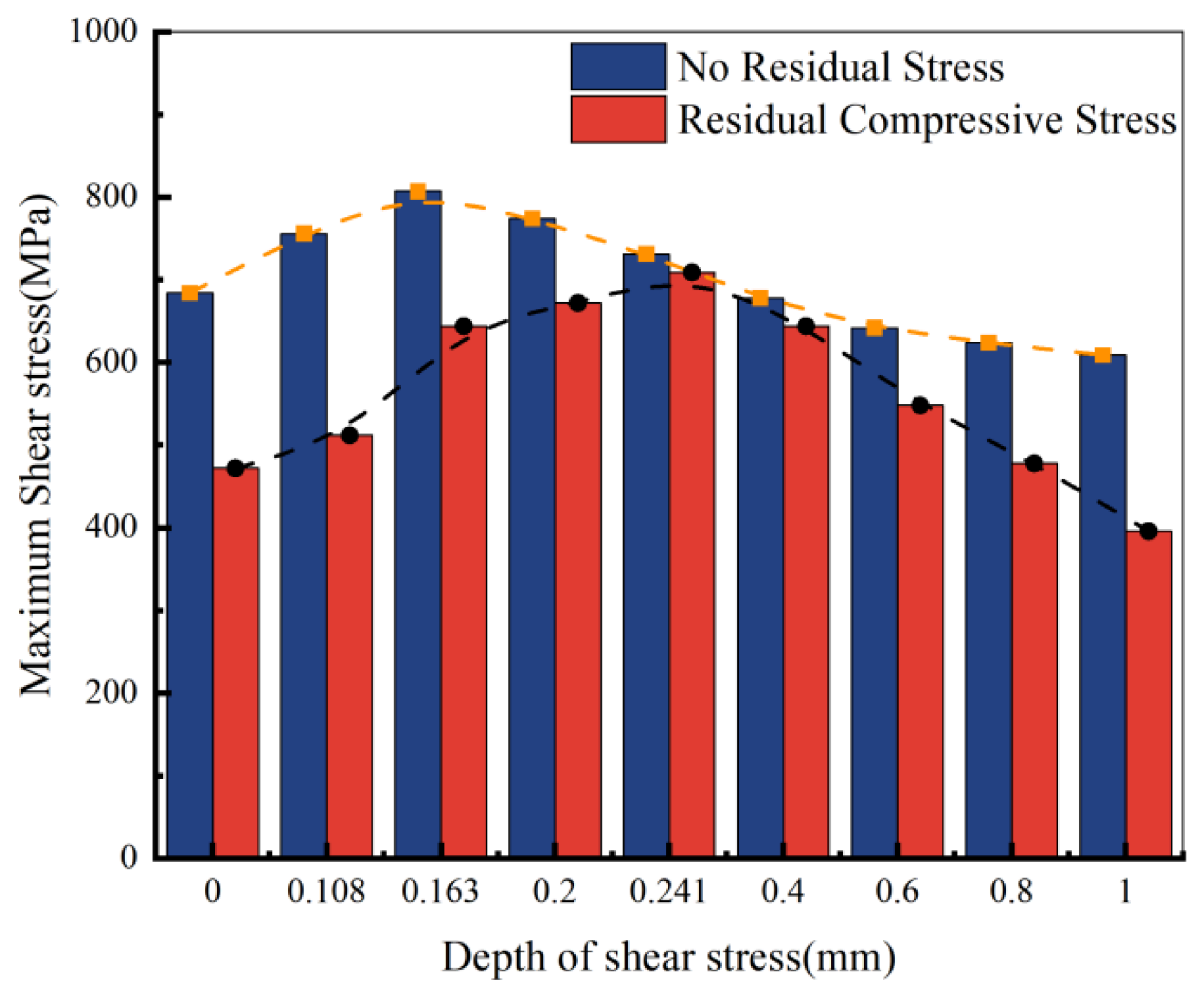
Maximum shear stress along the depth direction.

The subsurface shear stress distribution was analyzed to evaluate the influence of RCS on the critical location for fatigue initiation. Figure 7 presents a contour plot of the shear stress, providing a qualitative overview of the stress field. For quantitative analysis, the shear stress variation along the depth path (into the subsurface) was extracted and plotted in Figure 8.

A comparison of the curves in Figure 8 reveals the significant impact of RCS. In the absence of RCS, the maximum shear stress occurs at a depth of 0.163 mm. When RCS is present, this critical depth shifts to 0.241 mm. This shift of 0.078 mm implies that micro-cracks are likely to initiate in a deeper material volume, which may exhibit greater resistance to crack propagation, substantially lengthening the path a crack must travel to reach the surface and cause spalling.

#### 3.2.2. Orientation of Maximum Shear Stress

**Figure 9:**
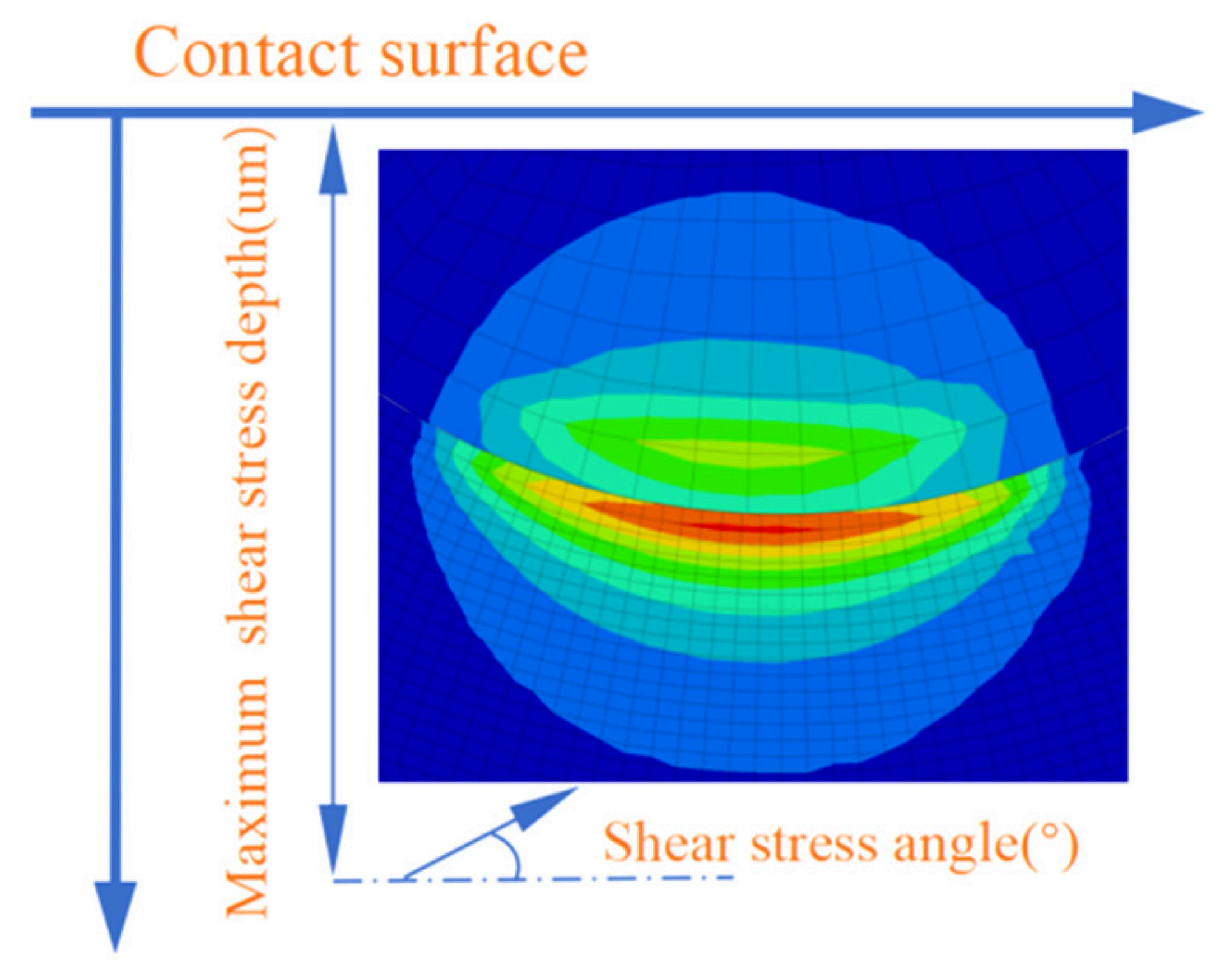
Schematic defining the inclination angle (θ) of the maximum shear stress plane.

**Figure 10:**
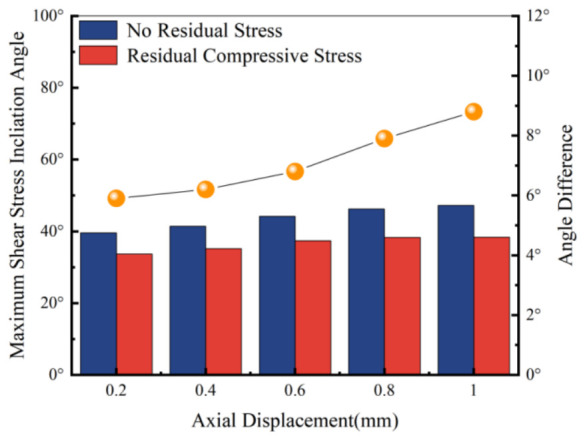
Inclination angle of the maximum shear stress plane with and without RCS.

The orientation of the maximum shear stress plane, which influences the initial crack path, was also investigated. Figure 9 defines the inclination angle (θ) of the maximum shear stress plane relative to the raceway surface. The effect of RCS on this angle is quantified in Figure 10.

As shown in Figure 10, RCS reduces the inclination angle by 9.72°. This alteration reorients the initial crack path to a direction less favorable for rapid propagation toward the surface. This change in crack initiation angle, combined with the increased depth, synergistically acts to decelerate the overall fatigue damage process, thereby enhancing the rolling contact fatigue life.

### 3.3. Impact of Friction Coefficient Under RCS on Contact Performance

#### 3.3.1. Contact Stress Under Varying Friction

**Figure 11:**
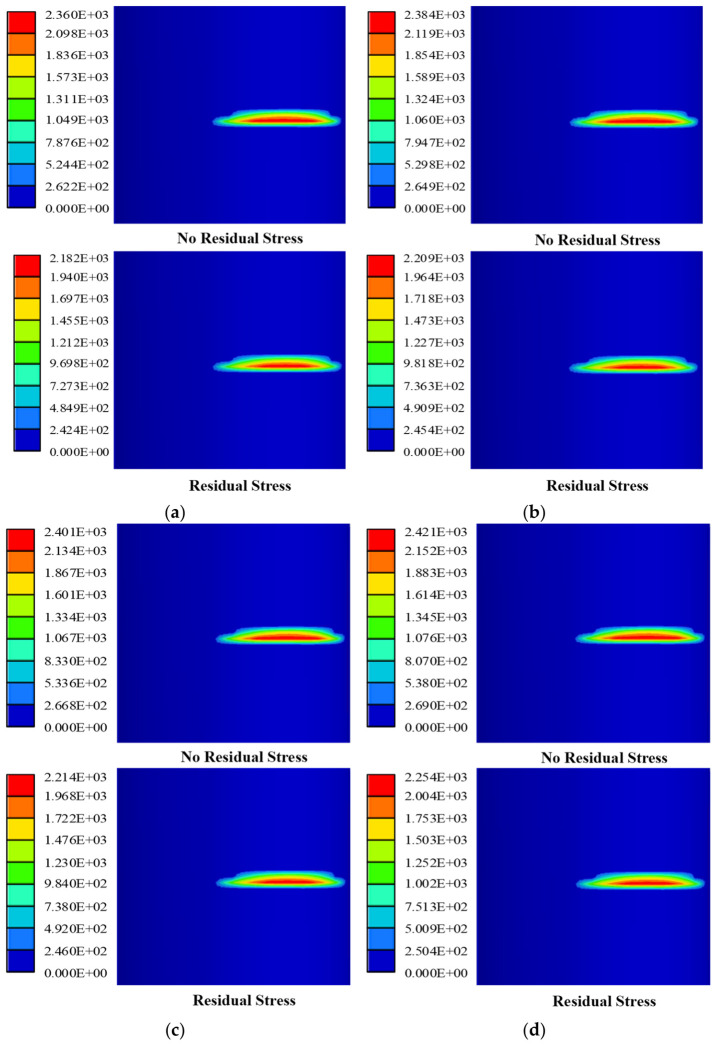

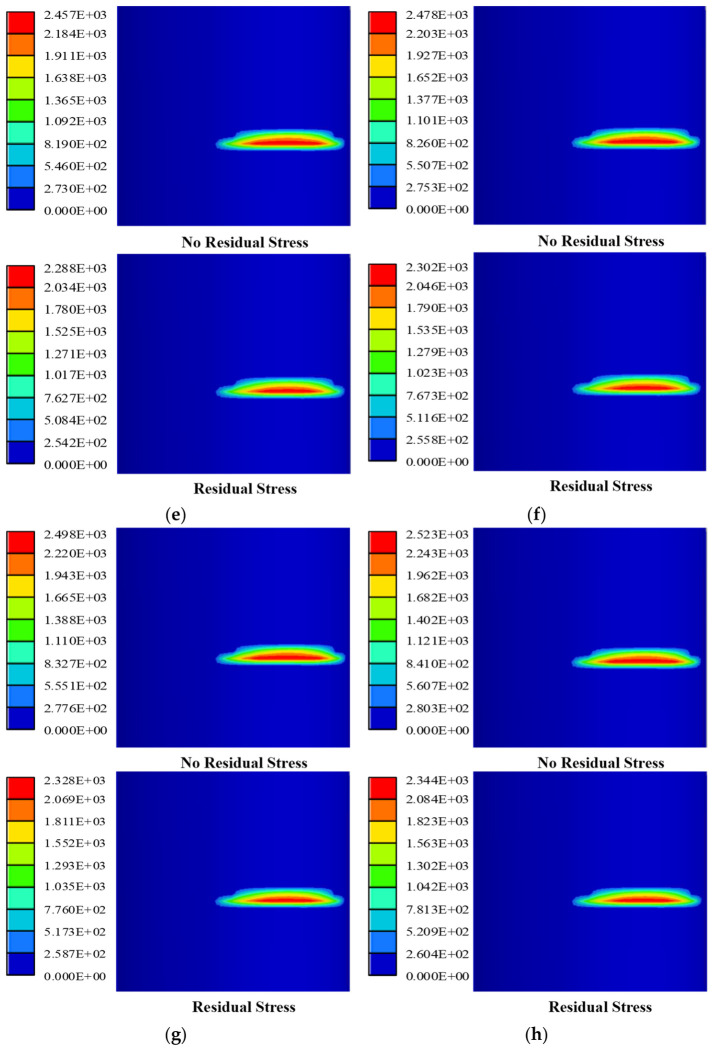
Contact pressure distribution (MPa) under different friction coefficients. (**a**) f = 0; (**b**) f = 0.005; (**c**) f = 0.02; (**d**) f = 0.05; (**e**) f = 0.1; (**f**) f = 0.15; (**g**) f = 0.2; (**h**) f = 0.25.

**Figure 12:**
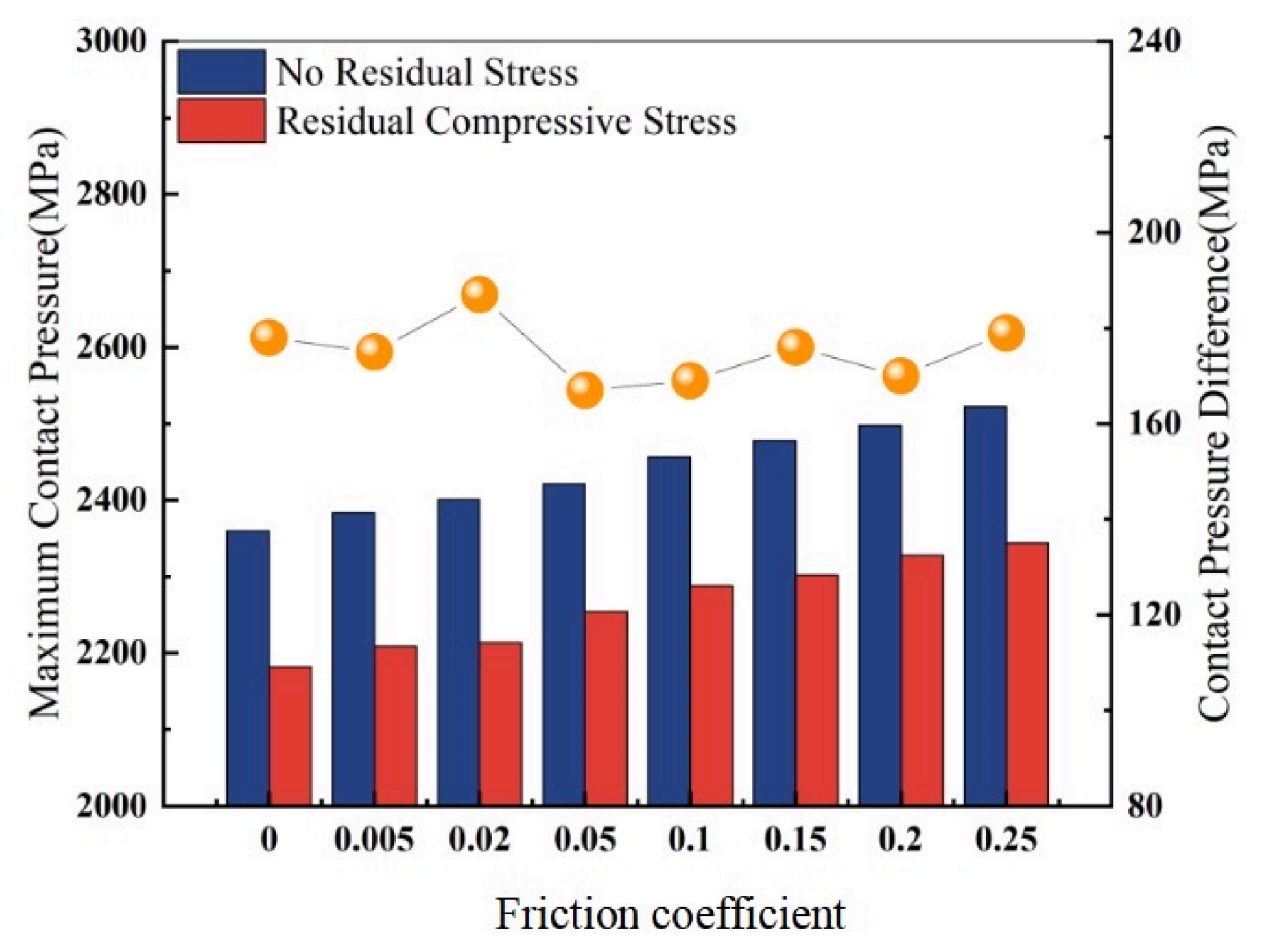
Maximum contact pressure under different friction coefficients.

The contact stress under different friction coefficients, with and without RCS, was analyzed with the inner ring subjected to a 1 mm axial displacement. Figure 11 illustrates the contact pressure distribution for a subset of the friction coefficients investigated, and Figure 12 plots the extracted maximum contact pressure against the friction coefficient.

As shown in Figure 12, the maximum contact pressure increases with the friction coefficient. This is because higher friction introduces significant shear tractions at the contact interface, which alter the stress field and lead to a stress concentration effect. Despite this increase, the presence of RCS consistently lowers the contact stress across the entire range of friction coefficients studied. On average, across the friction coefficients from 0 to 0.25, RCS reduces the maximum contact pressure by 178 MPa, which corresponds to a reduction of approximately 8.9% relative to the no-RCS case at a given friction level. This demonstrates the robust beneficial effect of RCS in mitigating contact severity, even under high-friction conditions.

#### 3.3.2. Maximum Shear Stress

**Figure 13:**
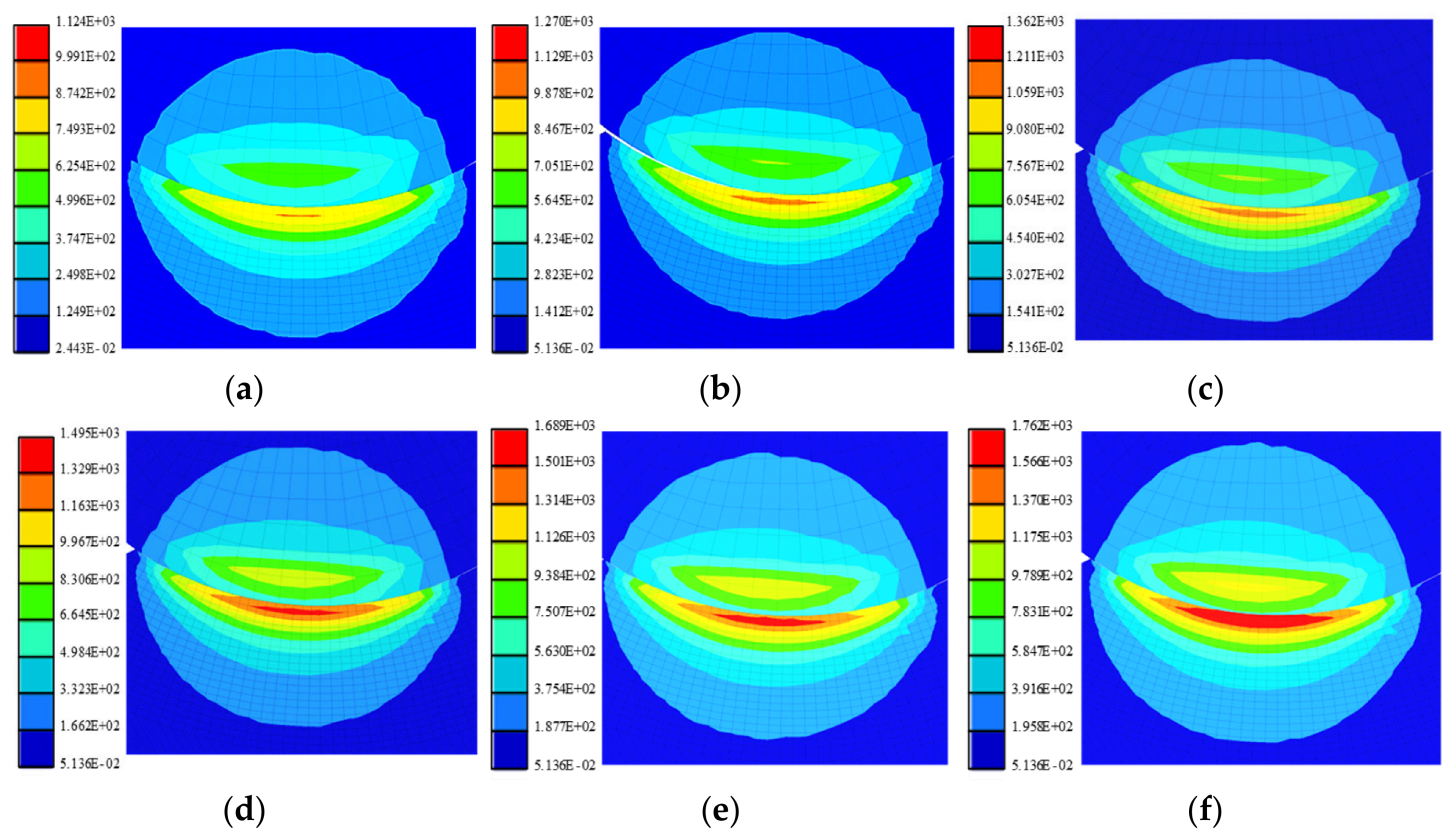
Subsurface shear stress distributions under different friction coefficients. (**a**) f = 0.005; (**b**) f = 0.02; (**c**) f = 0.05; (**d**) f = 0.1; (**e**) f = 0.15; (**f**) f = 0.2.

**Figure 14:**
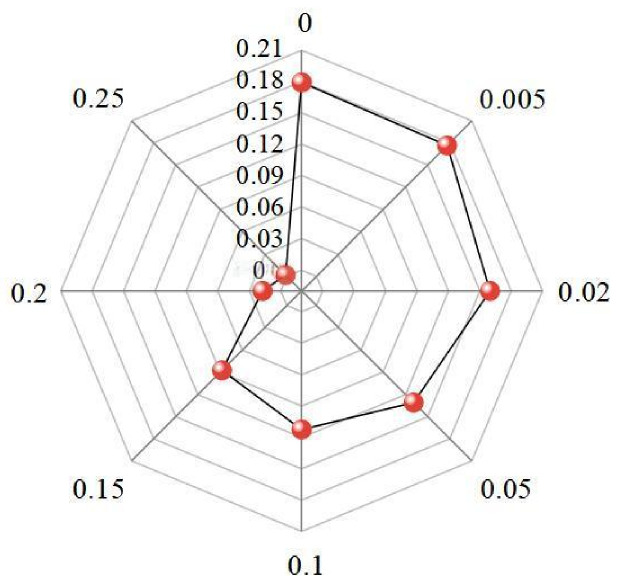
Depth of the maximum shear stress under different friction coefficients.

The depth of the maximum shear stress exhibits a strong dependence on the friction coefficient, as visualized in the contour plots of Figure 13 and quantified in Figure 14. As the friction coefficient increases, the critical depth decreases markedly, reaching a mere 0.00109 mm at a value of 0.25.

This extreme shallowing is attributed to the growing dominance of surface shear tractions, which intensify near-surface stress concentrations. As the maximum shear stress is driven to the surface, the dominant failure mechanism transitions from classical subsurface-initiated pitting to surface-driven damage, such as micro-pitting or wear. Consequently, the primary beneficial role of RCS in this high-friction regime is redefined: it primarily acts to mitigate the surface contact pressure (as shown in Section 3.3.1), thereby effectively delaying the onset of surface-related failures.

### 3.4. Impact of RCS Depth on Bearing Contact Performance

**Figure 15:**
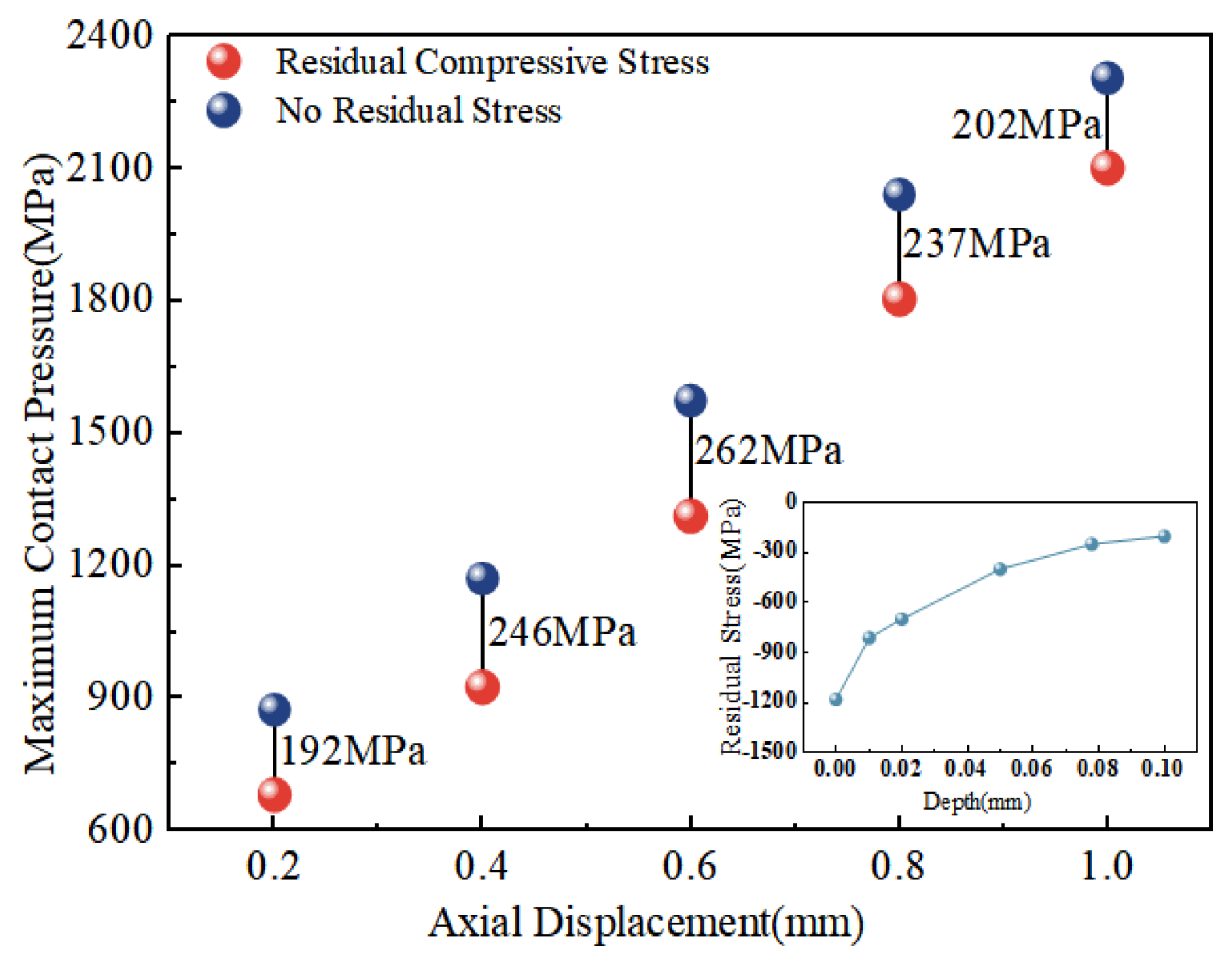
Maximum contact pressure with and without the 0.1 mm deep RCS layer.

**Figure 16:**
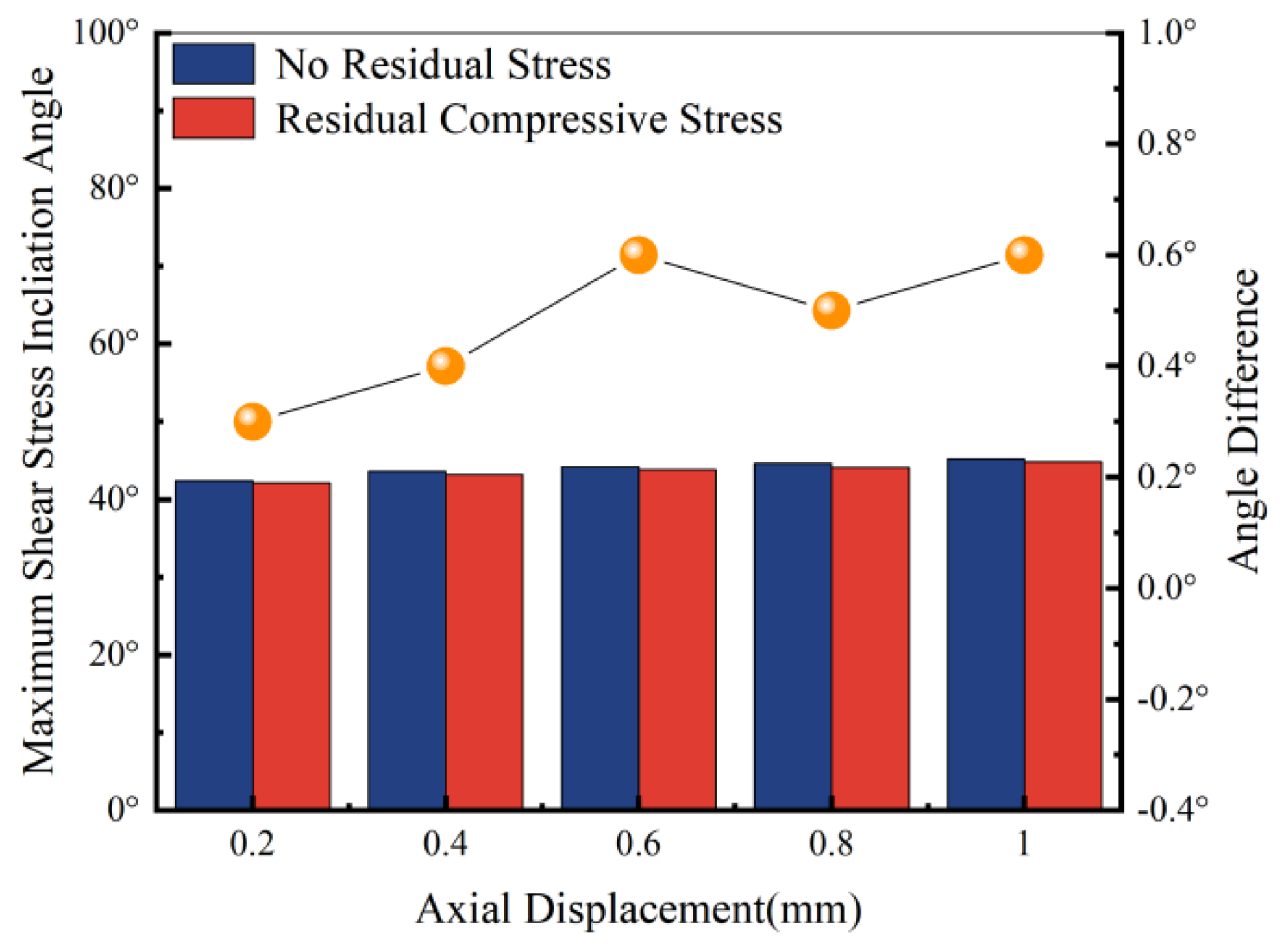
Maximum shear stress with and without the 0.1 mm deep RCS layer.

We also investigated the influence of a shallow RCS layer, with a depth of 0.1 mm, on the contact pressure and maximum shear stress. The results are shown in Figure 15 and Figure 16.

Under a 0.6 mm axial displacement, this shallow RCS layer effectively reduced the contact stress by 262 MPa. However, it had a negligible effect on the magnitude and depth of the maximum shear stress. This limited influence is explained by the location of the critical subsurface stress: for the present bearing under these loads, the maximum shear stress peak is situated at a depth of 0.16–0.24 mm (as established in Figure 8), which lies beyond the influence zone of the 0.1 mm deep RCS layer.

This result leads to a key conclusion: to effectively shield the material volume where fatigue cracks typically initiate, the compressive stress layer must possess sufficient depth to encompass the critical subsurface stress field.

### 3.5. Impact of Residual Stress on Bearing Fatigue Life

The fatigue life was calculated using the modified reference life method for universally loaded bearings (ISO/TS 16281:2008 [38]), which accounts for the actual load distribution. The core logic involves comparing the equivalent dynamic load the bearing experiences (influenced by RCS) to its intrinsic basic dynamic load rating. The procedure, defined by Equations (2)–(7), was applied identically to cases with and without RCS.

The fatigue life calculation is based on the ISO/TS 16281 standard, but RCS will change the crack initiation depth and crack propagation direction. Since these mechanisms are not included in the adopted life model, the life extension calculated in this paper fully reflects the actual physical impact of RCS.

(1) Determine basic dynamic load rating (fixed parameters)

The basic dynamic load ratings for the inner and outer rings, Q_ci_ and Q_ce_, are constants determined by the bearing’s geometry and material, calculated from Equations (2) and (3) based on the catalog rating C_r_.

For the inner ring, the Q_ci_ of single-row and multi-row bearings can be determined using the radial basic dynamic load C_r_:

(2) $$Q_{\mathrm{ci}}\;=\;\frac{C_{r}}{0.407Z(\cos\alpha )i^{0.7}}{(1\;+{\;\chi }^{10/3})}^{3/10}$$

For the outer ring, the Q_ce_ of single-row and multi-row bearings can be determined using the radial basic dynamic load C_r_:

(3) $$Q_{\mathrm{ce}}\;=\;\frac{C_{r}}{0.389Z(\cos\alpha )i^{0.7}}{(1\;+\;\chi^{-10/3})}^{3/10}$$

(4) $$\chi \;=1.044{\left(\frac{1-\gamma }{1+\gamma }\right)}^{1.72}{[\frac{r_{i}}{r_{e}}(\frac{{2r}_{e}-D_{w}}{{2r}_{i}-D_{w}})]}^{0.41}$$

(2) Calculate equivalent dynamic load from FEA results (variable inputs)

This is the critical step linking the finite element analysis to the life model. The equivalent dynamic loads Q_ei_ and Q_ee_ are variables that depend directly on the load distribution.

The FE model from Section 2.1 was solved for the conditions in Table 3.

The output is the contact force on each rolling element Q_j_, which is reduced by the presence of RCS.

**Figure 17:**
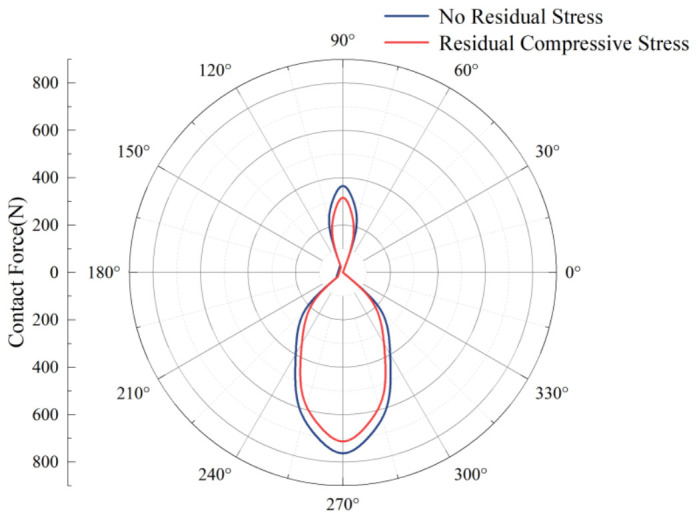
The outer ring contact load distribution diagram.

**Figure 18:**
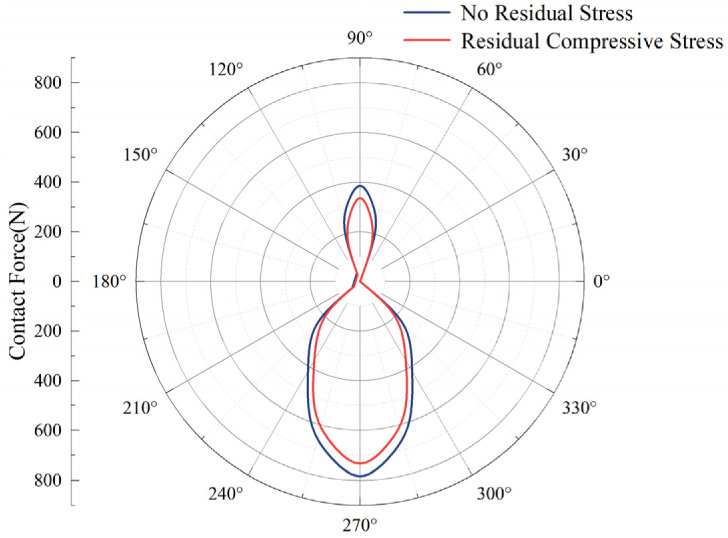
Inner ring contact load distribution diagram.

These force distributions (shown in Figure 17 and Figure 18) are the direct inputs for Equations (5) and (6).

For the inner ring, the rolling element equivalent dynamic load Q_ei_ of the inner ring that rotates relative to the bearing load is defined as follows:

(5) $$Q_{\mathrm{ei}}\;=\;{\left(\frac{1}{Z}\sum_{j=1}^{Z}{Q_{j}}^{3}\right)}^{1/3}$$

For the outer ring, the rolling element equivalent dynamic load Q_ee_ of the stationary outer ring or seat ring that rotates relative to the bearing load is defined as follows:

(6) $$Q_{\mathrm{ee}}\;=\;{\left(\frac{1}{Z}\sum_{j=1}^{Z}{Q_{j}}^{10/3}\right)}^{3/10}$$

(3) Calculate bearing life

The basic rating life L_10r_ (in millions of revolutions) and the life in hours L_h_ are calculated using Equations (7) and (8).

(7) $$L_{10r}={\left[{\left(\frac{Q_{\mathrm{ci}}}{Q_{\mathrm{ei}}}\right)}^{-10/3}+{\left(\frac{Q_{\mathrm{ce}}}{Q_{\mathrm{ee}}}\right)}^{-10/3}\right]}^{-9/10}$$

(8) $$L_{h}=\frac{10^{6}}{60n}L_{10r}$$

The parameters in the formula are defined as follows. C_r_ is the radial basic dynamic load rating [N]. D_w_ is the nominal ball diameter [mm]. Z is the number of rolling elements. Q is the rolling element load [N]. Q_ci_ is the rolling element load corresponding to the basic dynamic load rating of the inner ring [N]. Q_ce_ is the rolling element load corresponding to the basic dynamic load rating of the outer ring [N]. Q_ei_ is the equivalent dynamic load on the inner ring [N]. Q_ee_ is the equivalent dynamic load on the outer ring [N]. Q_j_ is the load on rolling element [N]. r_i_ is the inner ring raceway groove radius [mm]. r_e_ is the outer ring raceway groove radius [mm]. α is the nominal contact angle.

**Figure 19:**
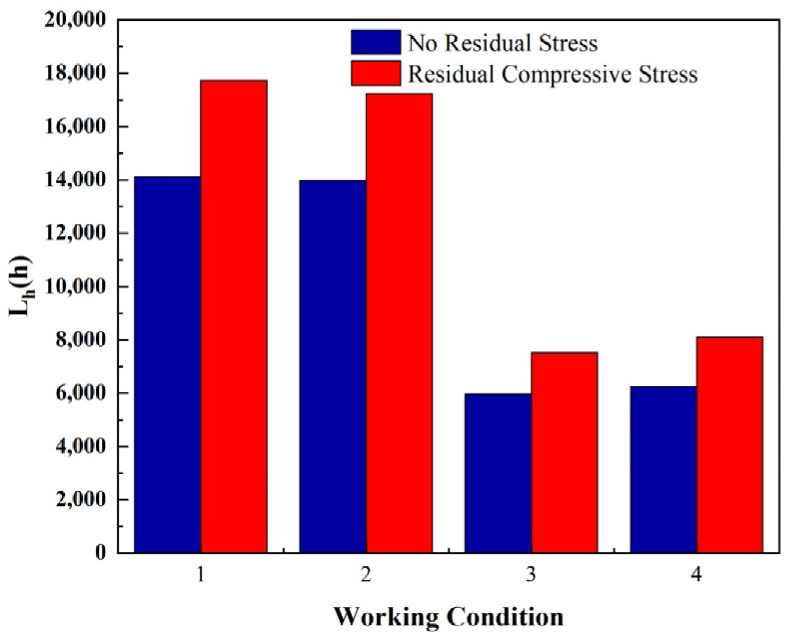
Comparison of calculated fatigue life.

Under the working conditions specified in Table 3, the application of RCS reduced the contact stress between the raceway and the ball. The resultant contact load distributions for the outer and inner rings are shown in Figure 17 and Figure 18, respectively. These reduced loads were used to compute the fatigue life, with the results summarized in Table 6 and Figure 19.

As illustrated in Table 7 and Figure 19, the introduction of RCS enhanced the fatigue life by 23.31% to 29.73%, based on the standard bearing life calculation. The variation in improvement across the tested conditions suggests a potential trend where the efficacy of RCS might be influenced by the loading state. The greatest relative benefit was observed under Condition 4, although a more extensive dataset would be required to conclusively establish this relationship.

It is important to note that this quantified life improvement is interpreted within the framework of the adopted model. The calculated enhancement is primarily attributed to the reduction in contact stress, as this is the dominant factor captured by the standard life theory. However, our finite element analysis has further revealed that RCS induces beneficial changes in the subsurface stress field—notably, an increase in the depth of the maximum shear stress and a decrease in its inclination angle, as detailed in Section 3.2.1 and Section 3.2.2. While these changes are mechanistically recognized to delay crack initiation and propagation—potentially contributing to additional life enhancement beyond the current calculation—they are not parameters in the present life prediction model. Consequently, the 23.31% to 29.73% life improvement calculated here should be viewed as a conservative estimate, stemming primarily from reduced contact stress. A more comprehensive life prediction model, integrating the beneficial changes in the subsurface stress field (increased maximum shear stress depth and reduced inclination angle), is a critical direction for future work and would likely predict even greater life extension.

In this paper, friction generates heat, causing the material temperature to increase and directly affecting bearing fatigue life. The study does not consider the influence of temperature on bearing fatigue life, so the research in this paper has certain limitations. Future work will consider the influence of temperature on the distribution of residual stresses, material properties and fatigue phenomena, in order to improve the accuracy of life prediction under actual working conditions.

## 4. Conclusions

This study investigated the mechanism by which RCS enhances the performance and fatigue life of ball bearings through a detailed finite element analysis. The main findings are summarized as follows:

**(1) Contact behavior improvement:** RCS significantly mitigates surface contact severity by offsetting a portion of the applied load. For the studied bearing under a 0.6 mm axial displacement, this mechanism reduced the maximum contact stress by 232 MPa and decreased the semi-major axis of the contact ellipse by 0.12 mm.

**(2) Subsurface stress field alteration:** A fundamental enhancement mechanism is the favorable modification of the subsurface stress field. RCS increased the depth of the maximum shear stress by 0.078 mm and decreased its inclination angle by 9.72°. These changes collectively impede crack initiation and delay propagation, thereby extending the fatigue life.

**(3) Depth-dependent efficacy:** The mechanistic role of RCS is depth-dependent. A shallow layer (e.g., 0.1 mm) effectively reduces contact stress (by up to 262 MPa) but has a minimal impact on the critical subsurface shear stress field. This highlights that a sufficiently deep compressive layer is crucial for optimal performance.

**(4) Overall life enhancement and outlook:** The application of RCS led to a substantial fatigue life improvement of 23.31% to 29.73%, as calculated by the standard model. It is important to note that this quantified improvement is derived from a model that primarily captures the benefit of contact stress reduction.

The beneficial alterations in the subsurface stress field identified in this study—the increased depth and reduced inclination angle of the maximum shear stress—are anticipated to contribute additional life extension. Therefore, future work should focus on developing a more comprehensive life prediction model that integrates these subsurface stress-state parameters alongside the contact stress. Such a model would provide a more accurate prediction of the fatigue life enhancement offered by surface treatments that induce RCS.

Future research will involve experimental verification, different residual stress distribution, and investigation into the effects of lubrication and other factors. It will also establish an improved fatigue life prediction model of the crack initiation mechanism.

## Figures and Tables

**Table 1:** Bearing material parameters.

Bearing Components	Elastic Modulus (Pa)	Poisson’s Ratio
Inner	2 × 10^11^	0.3
Outer	2 × 10^11^	0.3
Ball	2 × 10^11^	0.3

**Table 2:** Bearing geometric parameters.

Parameter	Values
Inner diameter d (mm)	496
Outer diameter D (mm)	542
Ball diameter D_w_ (mm)	13.4698
Number of balls	103
Bearing width B (mm)	28
Contact Angle α (°)	24

**Table 3:** Operational conditions for model validation.

Condition	F_z_ (N)	M_y_ (N·m)
1	17,079	3433
2	19,191	3648
3	51,449	18,519
4	56,593	15,934

**Table 4:** Comparison of contact pressure between FEA and theoretical analysis.

Condition	Contact Pressure (FEA) [MPa]	Contact Pressure (TA) [MPa]	Error [%]
1	1942	1987	2.3
2	2142	2204	2.4
3	3842	3901	1.5
4	3604	3699	2.5

**Table 5:** Mesh sensitivity analysis results (Condition 1).

Mesh Sizes (mm)	Contact Pressure (MPa/FEA)	Solution Time (h)	GCI
0.05	1943	3.5	4.5%
0.1	1942	1.5	4.6%
0.2	1942	0.5	4.5%
0.4	2164	0.2	5.2%
0.6	2238	0.1	5.3%

**Table 6:** Axial displacement loading conditions.

Case	1	2	3	4	5
Displacement (mm)	0.2	0.4	0.6	0.8	1

**Table 7:** Fatigue life comparison with and without RCS.

Condition	L_h_ (no RCS) [Hours]	L_h_ (with RCS) [Hours]	Life Increase [Hours]	Improvement [%]
1	14,120	17,742	3622	25.65
2	13,984	17,243	3260	23.31
3	5966	7524	1558	26.11
4	6242	8098	1856	29.73

## Data Availability

The original contributions presented in this study are included in the article. Further inquiries can be directed to the corresponding author.
